# An Efficient Algorithm for Partial Discharge Localization in High-Voltage Systems Using Received Signal Strength

**DOI:** 10.3390/s18114000

**Published:** 2018-11-16

**Authors:** Umar F. Khan, Pavlos I. Lazaridis, Hamd Mohamed, Ricardo Albarracín, Zaharias D. Zaharis, Robert C. Atkinson, Christos Tachtatzis, Ian A. Glover

**Affiliations:** 1 Department of Engineering & Technology, University of Huddersfield, Huddersfield HD1 3DH, UK; Hamd.ahmed@hud.ac.uk (H.M.); I.a.glover@hud.ac.uk (I.A.G.); 2 Departmento de Ingeniería Eléctrica, Electrónica, Automática y Física Aplicada, Escuela Técnica Superior de Ingeniería y Diseño Industrial (ETSIDI), Universidad Politécnica de Madrid, Ronda de Valencia 3, Madrid 28012, Spain; ricardo.albarracin@upm.es (R.A.); 3 Department of Electrical and Computer Engineering, Aristotle University of Thessaloniki, 54124 Thessaloniki, Greece; zaharis@auth.gr (Z.D.Z.); 4 Department of Electronic and Electrical Engineering, University of Strathclyde, Glasgow G1 1XW, UK; robert.atkinson@strath.ac.uk (R.C.A.); christos.tachtatzis@strath.ac.uk (C.T.)

**Keywords:** field trials, localization algorithm, least squares algorithm, partial discharge, ratio and search algorithm, RSS

## Abstract

The term partial discharge (PD) refers to a partial bridging of insulating material between electrodes that sustain an electric field in high-voltage (HV) systems. Long-term PD activity can lead to catastrophic failures of HV systems resulting in economic, energy and even human life losses. Such failures and losses can be avoided by continuously monitoring PD activity. Existing techniques used for PD localization including time of arrival (TOA) and time difference of arrival (TDOA), are complicated and expensive because they require time synchronization. In this paper, a novel received signal strength (RSS) based localization algorithm is proposed. The reason that RSS is favoured in this research is that it does not require clock synchronization and it only requires the energy of the received signal rather than the PD pulse itself. A comparison was made between RSS based algorithms including a proposed algorithm, the ratio and search and the least squares algorithm to locate a PD source for nine different positions. The performance of the algorithms was evaluated by using two field scenarios based on seven and eight receiving nodes, respectively. The mean localization error calculated for two-field-trial scenarios show, respectively, 1.80 m and 1.76 m for the proposed algorithm for all nine positions, which is the lowest of the three algorithms.

## 1. Introduction

Equipment reliability in transmission and distribution of electricity in HV systems has a pivotal role and has been a serious issue in the past [1]. The presence of PD in HV systems does not lead to instant failures. When PD occurs in an HV system, it causes a gradual degradation. PD becomes more damaging when it occurs in microscopic insulation voids [2]. This happens due to a microscopic void having a lower permittivity than its surrounding insulation, which results in an electric field in the void is higher than an electric field in its surrounding material. The electric field inside the void is usually higher than in the outside material, which results in a discharge occurring inside the void [3,4].

Issues such as manufacturing defects, poor repairs, poor quality, poor design as well as the aging processes can all lead to partial discharge [3,5]. PD only partially bridges the electrodes that sustain the electric field and it is thus a localized discharge. The PD pulse rise time is usually very low and the pulse usually lasts for 10ns [3,6,7]. PD pulses have a frequency spectrum in the high frequency (HF), very-high frequency (VHF) and the ultra-high frequency (UHF) range, a large amount of which is radiated from conductors that are in the close vicinity of the PD source.

PD activity mainly takes place in HV systems that include power generators, power transformers, power cables and switchgears [8]. Major sites where PD activity is heavy include cavities, joints, voids and delamination zones in HV systems [9,10,11].

To monitor an HV system’s state, PD detection surveys are usually performed on a periodic basis. Power companies normally measure PD activity every few months. The frequency of measurements is typically twice a year or not more than once every quarter. To continuously monitor and locate a PD activity, a novel location algorithm is proposed that is based on the received signal strength (RSS). The proposed algorithm locates the PD source in an anonymous environment, i.e., there is no prior information about the source transmitted power and the path loss exponent (PLE). The distance between two sensors is estimated from measured values of the received signal and localization is performed by using the multilateration technique. The algorithm is based on the propagation equation given as Equation (1) below:

(1) $$P_{R}=P_{t}-10\alpha log\left(\frac{r_{i}}{r_{1}}\right)$$

$P_{R}$ is the measured signal strength by the receiving node in d*Bm*, $P_{t}$ is the transmitted power of the source which is unknown and again measured in d*Bm*, α is the path loss exponent which again is unknown, however it can be constrained. $r_{i}$ and $r_{1}$ are the ith and the first node distance from the PD source in meter. The first node is chosen as the reference node hence $r_{1}$ represents the reference node distance from the PD source.

Practically, Equation (1) remains unsolvable due to $P_{t}$ and α being unknown and this is the main challenge to be resolved to estimate the location of the unknown source. Firstly, source transmitted power is eliminated by using the ratio of distances approach which is explained in detail in Section 2. Secondly, to overcome with PLE issue, an initial value of PLE is chosen and mean spatial location is estimated from all estimated locations. The path loss exponent is then optimized within constrained limit. This is again explained in detail in Section 2. The feasibility of the algorithm was tested by estimating the location of PD source at nine different positions. Comparisons of the proposed algorithm with the ratio and search and the least squares algorithms show that the proposed algorithm offers better accuarcy for at least the two field-trials that were performed.

Section 1.1 describes briefly about existing algorithms for PD localization and PD signal radiation. Section 2 mainly explains details about the proposed algorithm and how the system of equations is solved when path loss parameters are unknown. Section 3 focuses on the experimental setup and results. Section 4 presents the conclusions.

### 1.1. Algorithms for Wireless PD Localization of PD Sources

The PD pulse phenomenon is random in nature. The quality of HV systems and cables can be assessed by measurement and diagnosis of PD. Various methods have been deployed in the past for the detection and localization of different PD types [5]. Lateration techniques used for PD localization are based on distance. PD source location based on spatially-separated sensors has been explored in the past by using various techniques including radiofrequency (RF) antenna array, time of arrival (TOA), time difference of arrival (TDOA), direction of arrival (DOA), use of SDR USRP N200 (from Ettus research, Santa Clara, CA, USA) and RTL-SDR (from Nooelec, New York, NY, USA) etc. [5,8,10,11,12,13]. Another interesting PD location mechanism proposed in [14] is based on advanced auscultatory technique uses the amplitude of the received signal to estimate the source location. In recent years, the radiometric RF detection of PD has gained significant popularity due to advancements in the field of communication engineering. The cost of hardware at HF, VHF and UHF operating bands has reduced significantly in recent times, which make it affordable to detect PD in these frequency bands [9,15,16,17]. All these methods are classified as range-based methods, i.e., they form matrices bearing location information and subsequently they estimate the position of the source based on the information held in location matrices.

Continuous monitoring of PD band-limited signal phenomena requires a real-time location system. Real-time location methods implemented in the past for mobile device positioning can be classified into lateration, angulation and pattern recognition [12,18]. PD localization accuracy is limited by the fact that PD pulse is time-limited, i.e., it has a certain rise-time. Owing to measurement systems limitations and propagation effects, the received RF signal will be a band-limited signal. This brings uncertainty in the time-of-flight of PD pulse and hence will cause inaccuracy in location measurement [19].

The RF antenna array method is based on the radiometric location of a PD source. A wideband RF interference is generated by PD, which can be intercepted by using the radio receiver. The work in [20] has used a 4-antenna array for three-dimensional localization of PD sources. The antenna array with direct sampling can measure the time of arrival of the wave to a nanosecond accuracy [21].

**Figure 1:**
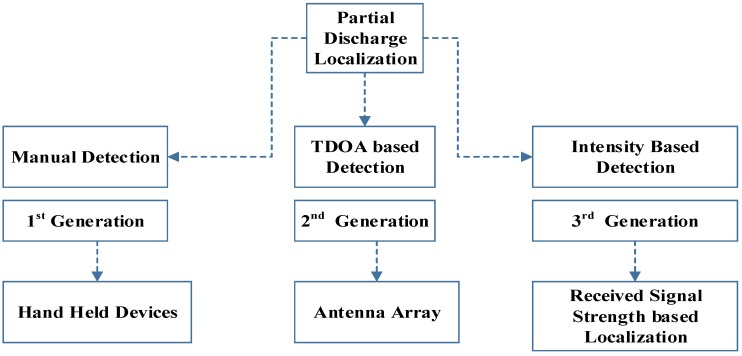
PD localization techniques.

In TOA, both transmission and receiver synchronizations are necessary [22,23]. In TDOA only receiver synchronization is necessary [24]. In both these schemes, a small inaccuracy can lead to significant location errors [25,26]. For multipath interference, it may lead to inaccuracies and hence may sometimes hardly be applicable in practice. In the RSS method, however, there is no synchronization between nodes is required, because the technique work on the received energy rather than the time bound PD pulse and this, therefore, enhances scalability. In all the three schemes above scalability remains the biggest constraint due to the synchronization requirement. However, on the other handside, the scalability also improves accuracy [27,28]. The RSS method is an energy detection method and is again based on a lateration approach like TOA and TDOA. Generally, and based on literature, PD localization systems can be summarized as shown in Figure 1.

## 2. RSS Localization Algorithm Description

The proposed algorithm is based on the path loss model Equation (1) given in Section 1. The equation has two unknowns as mentioned above, i.e., source transmitted power ($P_{t}$) and the path loss exponent (α). Due to this, Equation (1) remains unsolvable.

Firstly, to overcome the source transmitted power issue, the source transmitted power is eliminated by using a ratio of distance approach. In ratio of distance approach, one of the nodes in the receiving system is chosen as a reference node. The distance of all other receivers in the receiving systems is divided by the distance of the reference node. In this way, the uncertainty of the source transmitting power is eliminated. Each node in the receiving system is used as the reference node in turn and a mean estimated location is estimated from all estimated locations for an initially chosen value of path loss exponent. To do this, Equation (1) is converted into distance by re-arranging it in the form of distance as given in Equation (2)

(2) $$r_{i}=r_{1}\left(10^{\frac{P_{t}-P_{R}}{10\alpha }}\right)$$

The coordinates of the receiver that receive the signal transmitted by the source are named as (*x_i_*, *y_i_*). The RMS distance between the source, and the ith receiver is given by Equation (3) below:

(3) $$r_{i}^{2}={\left(x_{i}-x\right)}^{2}+{\left(y_{i}-y\right)}^{2}$$

Then, Equation (2) can be simplified as shown in Equations (4) and (5) below:

(4) $$p_{i}=10^{\frac{P_{R}}{10}}$$

(5) $$p_{1}=10^{\frac{P_{t}}{10}}$$

Equation (2) using Equations (4) and (5) is compared with Equation (3) as shown in Equation (6) below:

(6) $${\left(x_{i}-x\right)}^{2}+{\left(y_{i}-y\right)}^{2}={\left(r_{1}{\left(\frac{p_{i}}{p_{1}}\right)}^{\frac{1}{\alpha }}\right)}^{2}$$

The distance ratio of the reference node to the ith node is given in Equation (7):

(7) $$\frac{r_{1}^{2}}{{\left(x_{i}-x\right)}^{2}+{\left(y_{i}-y\right)}^{2}}={\left(\frac{p_{i}}{p_{1}}\right)}^{\frac{2}{\alpha }}$$

The distance $r_{1}$ of the reference node from the PD source is given in Equation (8):

(8) $$r_{1}=\;\sqrt{{\left(x_{1}-x\right)}^{2}+{\left(y_{1}-y\right)}^{2}}$$

Equation (7) in the ratio form is given in Equation (9):

(9) $$\frac{{\left(x_{1}-x\right)}^{2}+{\left(y_{1}-y\right)}^{2}}{{\left(x_{i}-x\right)}^{2}+{\left(y_{i}-y\right)}^{2}}={\left(\frac{p_{i}}{p_{1}}\right)}^{\frac{2}{\alpha }}$$

By cross multiplying Equation (9), expanding the square and by rearranging all terms in the form of x, y and z, where $z=\;x^{2}+y^{2}$ is an extra variable, a system of matrices in the form of AX=b is obtained. The coefficients of x, y and z for i=2, i.e., the second node in the receiver system are shown respectively, in Equations (10)–(12):

(10) $$x=\;2p_{2}^{\frac{2}{\alpha }}x_{2}-2p_{1}^{\frac{2}{\alpha }}x_{1}$$

(11) $$y=2p_{2}^{\frac{2}{\alpha }}y_{2}-2p_{1}^{\frac{2}{\alpha }}y_{1}$$

(12) $$z=p_{1}^{\frac{2}{\alpha }}-p_{2}^{\frac{2}{\alpha }}$$

If there are m receivers used to receive the signal, the matrix representation of the whole system is shown respectively, in Equations (13)–(15). 

The algorithm requires at least four nodes and one of the nodes will be used as the reference node. To enhance accuracy, the number of receiving nodes can increase to as many as fulfil the accuracy requirements. This means that multiple equations will be established i.e., an equation for each receiving node in the system, and a matrix form equation will be formed. The relevant matrices are shown below:

(13) $$A=[\begin{array}{ccc} 2p_{2}^{\frac{2}{\alpha }}x_{2}-2p_{1}^{\frac{2}{\alpha }}x_{1} & 2p_{2}^{\frac{2}{\alpha }}y_{2}-2p_{1}^{\frac{2}{\alpha }}y_{1} & p_{1}^{\frac{2}{\alpha }}-p_{2}^{\frac{2}{\alpha }}\\ 2p_{3}^{\frac{2}{\alpha }}x_{3}-2p_{1}^{\frac{2}{\alpha }}x_{1} & 2p_{3}^{\frac{2}{\alpha }}y_{3}-2p_{1}^{\frac{2}{\alpha }}y_{1} & p_{1}^{\frac{2}{\alpha }}-p_{3}^{\frac{2}{\alpha }}\\ \vdots & \vdots & \vdots \\ 2p_{m}^{\frac{2}{\alpha }}x_{m}-2p_{1}^{\frac{2}{\alpha }}x_{1} & 2p_{m}^{\frac{2}{\alpha }}y_{m}-2p_{1}^{\frac{2}{\alpha }}y_{1} & p_{1}^{\frac{2}{\alpha }}-p_{m}^{\frac{2}{\alpha }} \end{array}]$$

(14) $$X=\left[\begin{array}{c} x\\ y\\ z \end{array}\right]$$

In Equation (14) x and y are the estimated coordinates of the source in meters:

(15) $$b=\;\left[\begin{array}{c} \begin{array}{c} p_{2}^{\frac{2}{\alpha }}-\;p_{1}^{\frac{2}{\alpha }}\\ p_{3}^{\frac{2}{\alpha }}-p_{1}^{\frac{2}{\alpha }}\\ \vdots \end{array}\\ p_{m}^{\frac{2}{\alpha }}-p_{1}^{\frac{2}{\alpha }} \end{array}\right]$$

The above system of equations represents matrix form of AX=b.

The system of equations shown in the above matrix for is over determined, i.e., the number of unknowns are less than the number of equations. To solve the above system, linear least squares approach has been used, based on Equation (16) [29]:

(16) $$X={\left(A^{T}A\right)}^{-1}A^{T}b$$

Secondly, the above expression cannot be solved yet. Although the source transmitted power is eliminated by taking the ratio of distances, however, the path loss exponent (α) is still unknown. A positive aspect about the path loss exponent is that it is constrained i.e., it has a practical minimum and maximum. Theoretically and experimentally, it has been proven that for an ideal free space propagation, α is approximately 2. However, considering the factors such as multipath propagation and shadowing, it ranges from 1≤α≤5 [30,31,32]. For this reason, the initially chosen value of PLE is 2 because the measurements are performed in an approximately free space environment in a large sports hall. The process is then repeated for multiple values of PLE by taking a reasonable step size, e.g., 0.01, keeping in view the runtime. A measure of the spread between the mean spatial location and the estimated location is calculated by using Equation (17) below:

(17) $$d_{RMS}=\sqrt{\frac{1}{N}\overset{N}{\underset{n=1}{\sum }}d_{n}^{2}}$$

where, $d_{n}$ is the spatial location distance from the mean estimated location in meters, and $d_{RMS}$ is the RMS spread of the spatial location distance in meters. The location that will have the minimum value of RMS spread will be the estimated location of the source, and the value of the path loss exponent will be an optimized value closest to an average PLE of the environment.The whole algorithm is summarised in the following steps:

- Assume a universal value of path loss exponent (α) from the given range 1≤α≤5.
- Select a reference node and use ratios of power received by a pair of sensors to calculate an estimated PD location.
- Repeat for all other nodes set as reference nodes one by one.
- Calculate mean spatial location from all the above estimated locations.
- Calculate the RMS error of spatial location distance from mean location.
- Repeat for multiple values of α and select the final estimated location that has a minimum RMS error.

**Figure 2:**
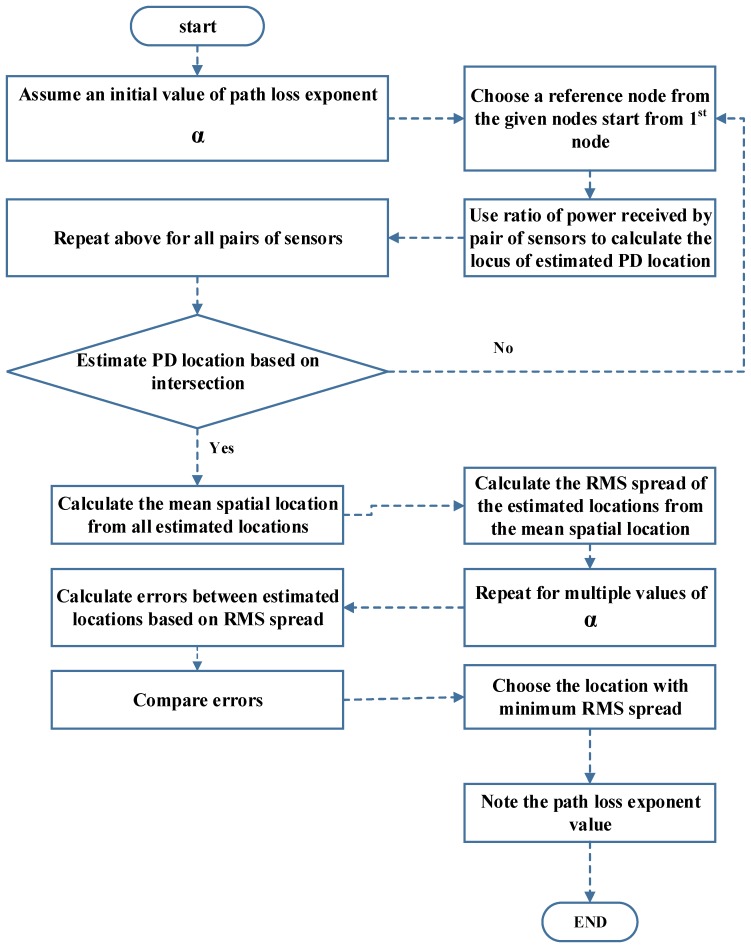
Algorithm flowchart.

The overall flowchart of the algorithm is shown in Figure 2.

## 3. Experimental Setup and Results

**Figure 3:**
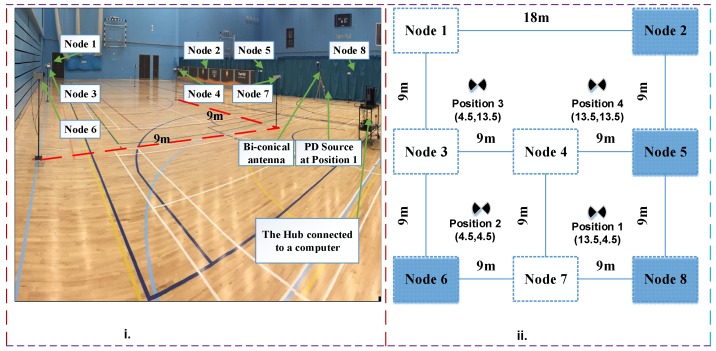
(**i**) Test space image. (**ii**) Sensors arrangement schematic.

Figure 3 shows the experimental set-up and the schematic. Figure 3i illustrates the experimental setup inside a sports hall. Figure 3ii illustrates the arrangement of sensors where four out of the nine positions of the source used are shown as examples. Due to space constraints sensors were kept at 9 m distance from each other. Sensors nodes communicate via the central hub using the wireless highway addressable remote transducer (wirelessHART) protocol.

To evaluate the performance of the algorithm, an offline PD signal was generated by using a commercial high voltage partial discharge (HVPD) calibrator (from HVPD Ltd., Salford, UK). The HVPD calibrator was selected due to its suitability for IEC 60270 standard measurements. It can provide a wide range of calibration pulses ranging from 1 pC to 100 nC and the pulse repetition rate is selectable at 100, 200 and 400 Hz.

**Figure 4:**
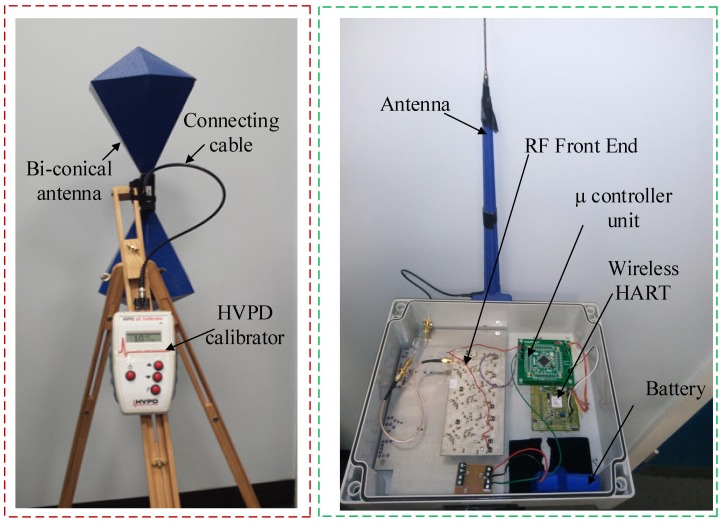
Biconical antenna with PD calibrator and the radiometric sensor used.

The calibrator was used to generate a 10 nC charge with a repetition rate of 100 Hz and it was connected to a biconical Aaronia Bicolog 20100 E radiating antenna (from Aaronia AG, Strickscheid, Germany). This (on the left) together with the RF radiometric sensor used (on the right) are shown in Figure 4.

Specialized PD signal emulators in [33] producing random PD signals have also been tested during this field-trial with very similar results as with the calibrator when proper averaging was applied to the received signal.

**Figure 5:**
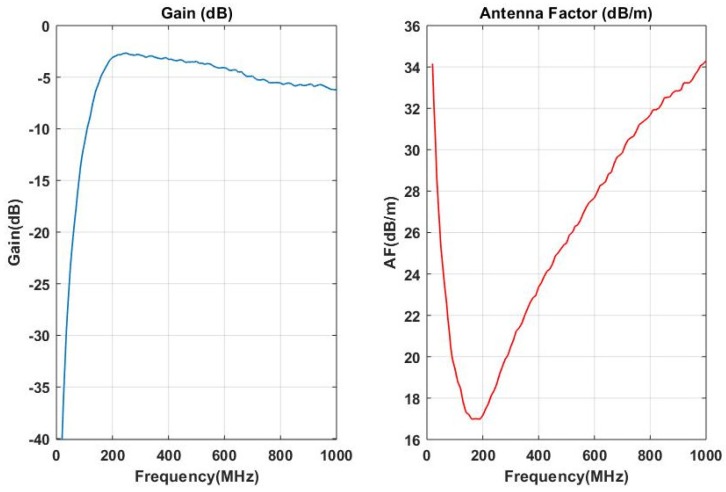
Antenna gain versus frequency and antenna factor plot.

The biconical antenna has dimensions  0.54 m ×0.225 m × 0.225 m, the frequency range from 20 MHz to 1 GHz and the input impedance of 50 Ω. The plot of frequency versus gain and the antenna factor of the antenna is illustrated in Figure 5 below:

To measure PD signal, eight receiving nodes were used. There were nine different measurements taken in a free space environment (indoors). Measurement sensors were placed over an 18 × 18 m grid. Measurements were performed in an unshielded sports hall environment. A range of services operate at the desired frequency band such as FM broadcast and digital video broadcasting (DVB-T), etc.

**Figure 6:**
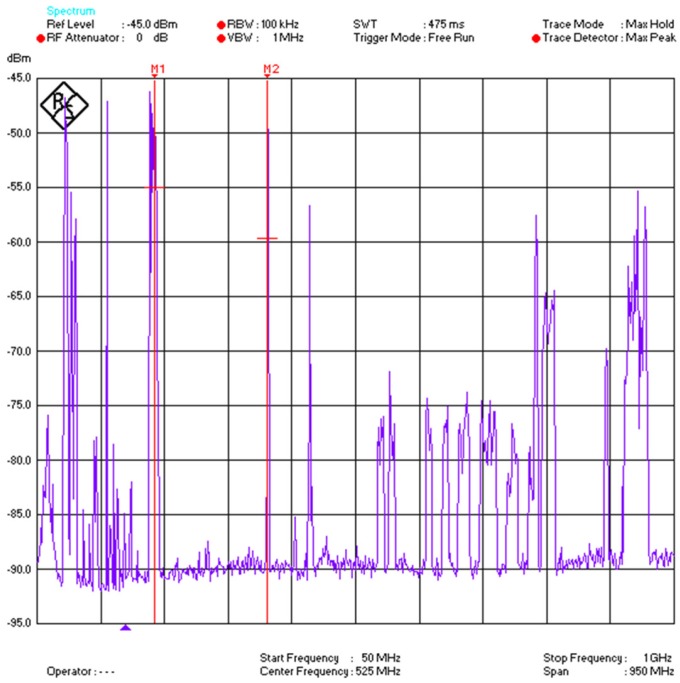
Spectral analysis inside the sports hall.

Signals for such services could easily superimpose on the desired PD signal and could become a source of noise to the desired signal. To evaluate such background interferences, a spectral analysis was performed inside the sports hall before measurements were conducted by using a high specification spectrum analyzer as shown in Figure 6 below. 

**Figure 7:**
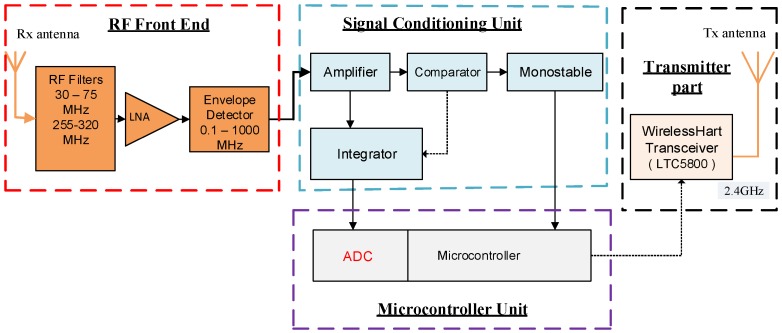
Block diagram of a PD signal measurement radiometer sensor system [34].

As illustrated in Figure 6, the frequency span was chosen from 50 MHz to 1 GHz covering the whole desired band. As it is evident from Figure 6, interference from FM radio, TV, LTE-4G, GSM and other communication signals were observed. To overcome such effects, bandpass RF filters have been used in the front-end part of the measurement sensor receiver as in Figure 7.

### 3.1. Measurement System

**Figure 8:**
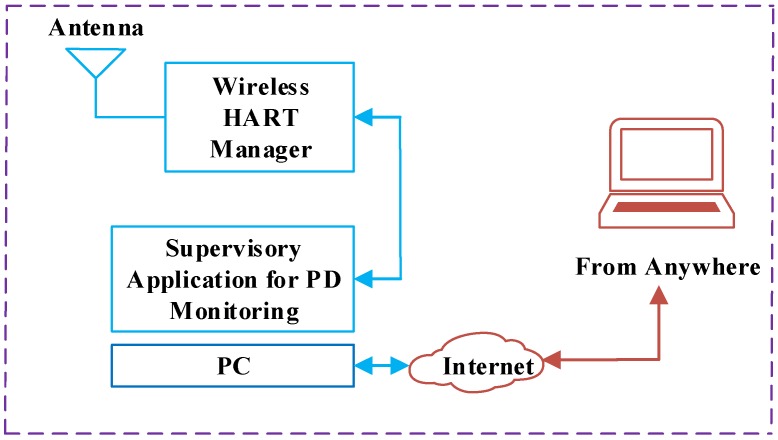
Overview of the supervisory system.

Figure 7 and Figure 8 show the RF measurement sensor and supervisory part, respectively.

Figure 7 is the radiometer sensor and Figure 8 is the supervisory system. Sensor nodes used for measurement consisted of four major sub-systems including the RF front end, signal conditioning, microcontroller and the wirelessHART unit. Such sensors are simple and cost-effective and can be deployed for continuous monitoring of PD.

The explanation about each part of the sensor system is given next.

#### 3.1.1. RF Front End

The RF front end consists of four components which include:

- Receiving dipole antenna
- RF bandpass filters
- Low noise amplifier (LNA)
- RF peak envelope detector

A dipole receiving antenna has been used to receive the PD signal that is emitted from the dielectric material. The dipole antenna has the vertical polarization with the frequency range from 20 MHz to 1 GHz with an omnidirectional response. Once received by the antenna, the signal is then passed to the RF filters. The experimental study suggests that PD signal bandwidth remains between 50–800 MHz, the used passbands have a frequency range from 30 to 75 MHz and 255 to 320 MHz [34,35]. To remove unwanted signals that could be present in the monitored signal from various sources such as TV, FM, digital radio and private radio, RF filters have been used at these two bands as shown in RF front-end part of Figure 7. 

The function of the LNA is to increase the sensitivity of the sensor by providing a fixed gain value of 16.5 dB. The use of RF filters and LNA enables RF front end part of the system to generate the frequency response in the range of 30 to 75 MHz and 255 to 320 MHz with the noise figure value from 5–7 dB and passband gain value of 12–14 dB. By doing this, a 20-m range is obtained from the PD source which is far above the minimum set requirement of 10 m. The reason to set the minimum 10-m range is to make the system cost effective and practically usable for continuous PD monitoring and localization. The function of the envelope detector is to reduce the signal bandwidth by removing the RF component and leaving envelope only.

#### 3.1.2. Signal Conditioning Unit

The output from the envelope detector is fed to the signal-conditioning unit where further amplification is performed via an amplifier, in addition to counting the PD events received. Within the signal-conditioning unit, the envelope-detected signal is integrated as well. The output from the signal conditioning unit is a collected PD activity in the form of metric. The threshold value is set to 3 V. When the output of the integrator reaches the threshold value; the integrator is set to zero. The function of the comparator is to activate the integrator, provided that the PD signal is of the sufficient value. This is vital to ensure that integrator’s output voltage is not a result of envelope detected noise signal. Another function of the comparator is to count the number of PD events received. The integrator will stop integrating once the signal strength drops below the threshold. At this point, the output of the integrator is kept at a constant level. The signal then is passed to the microcontroller unit.

#### 3.1.3. Microcontroller Unit

From the signal conditioning unit, two parameters are received by the microcontroller unit which includes:

- The step size of the integrator
- Received PD pulses count

The function of the microcontroller within this entire system is to provide the interface between the sensor and the wirelessHART unit. The microcontroller used is PIC24EP512GP810 (from Microchip, Chandler, AZ, USA). The microcontroller has the random access memory (RAM) of 52 KB, the program memory of 512 KB and speed of 70 million instructions per second (MIPS). The microcontroller unit offers analogue to digital conversion (ADC) that is configurable as 10 bits with 1.1 mega samples per second (MSa/s). There are four simultaneous channels are provided that ensure that sampling of the PD pulses is performed adequately.

The second important function of the microcontroller unit is to establish and maintain the wireless connection via the wirelessHART unit. Microcontroller is brought to the sleep mode if there is no data collection to save energy. When the signal is received via a supervisory application, the microcontroller unit wakes up from the sleep mode. The data is collected and it is transferred to the system. PD occurrences are monitored by the microcontroller for one second, i.e., 50 cycle of the power supply. During this one second, three main tasks are performed which include, the counting of PD pulses, sampling of integrator step size and recording of the relative time stamp of PD pulses. The data is then transferred to the supervisory application via the wirelessHART unit after calculating the average step size. The whole process is repeated on an hourly basis.

#### 3.1.4. WirelessHART Transceivers

To continuously monitor PD, it is pivotal to have robust interfacing of PD sensors to the wireless network. For this reason, WirelessHART IEC 62591 has been used as wireless communication technology. Wireless HART provides a continuous PD monitoring with the option of scalability if the scope of the system deployment gets bigger. It is a low power, low cost and easy to install communication system that is based on IEEE 802.15.4. It is a self-forming multi-hop mesh technology. The technology is specifically designed for harsh industrial environments.

#### 3.1.5. Supervisory Application for PD Monitoring

Supervisory application for PD monitoring has three main parts, which include:

- A data collection module
- A monitoring module
- A location algorithm

The data collection module collects data by interacting with a wirelessHART unit and stores it into a database. The monitoring module is a database system that is based on Indusoft web studio. The full details are described in [36]. The location algorithm uses the received signal and estimates the location of the PD source.

To obtain the measurements, eight measurement nodes have been used and arranged as shown in Figure 6. Each measurement sensor is an individual radiometer. The locations of the measurement sensors were kept the same and the location of the source was changed.

The received signal was in millivolts (mV) that was converted into d*Bm*. The average-step voltage signal was converted into d*Bm* as an input to the location algorithm. The signal was converted as below:

(18) $$dBm=20log_{10}\left(U^{2}\right)+30$$

and is shown in Table 1 for each node and all positions.

Table 1 shows the values in d*Bm* for each position of the source for each node. For each position, several measurements were performed and Table 1 shows the average of all measurements performed. From the measurements, it is clear that receiving nodes closer to the source have higher signal strength than the ones that are far from the source. For example, position 1 (13.5, 4.5) has the closest nodes 4, 5, 7 and 8. All four nodes have the strongest received signal than other nodes in the receiving system. Nodes 1 and 2 that are farthest have the least strength of the received signal. PD source localization was performed by using two scenarios:

● Scenario 1: PD source localization using seven sensors

In Scenario 1, seven measurement sensors were used and PD source location was estimated for all nine positions. Table 2 shows the estimated location, error and path loss exponent.

**Figure 9:**
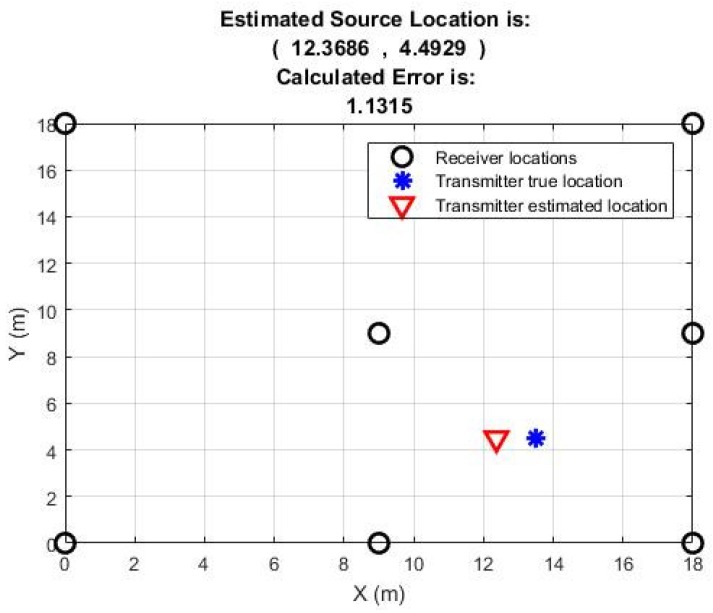
Position 1 result of the estimated source location with seven receiving nodes.

Table 2 shows results for the estimated location as well as the optimum path loss exponent which is from 1.60 to 3.45 for the majority of the positions with an exception for position 4 where it is 4.25 m. The calculated error is reasonably low for the majority of the positions as well. For two positions it is less than one meter, for four positions it is between 1 to 2 m and for three positions it is more than 2 m. Estimated location for position 1 when seven measurement nodes were used is shown in Figure 9.

● Scenario 2: PD source localization by using eight sensors

When eight measurement sensors were used, the estimated coordinates of the source, error calculations and optimum value of path loss exponent for each position are shown in Table 3.

**Figure 10:**
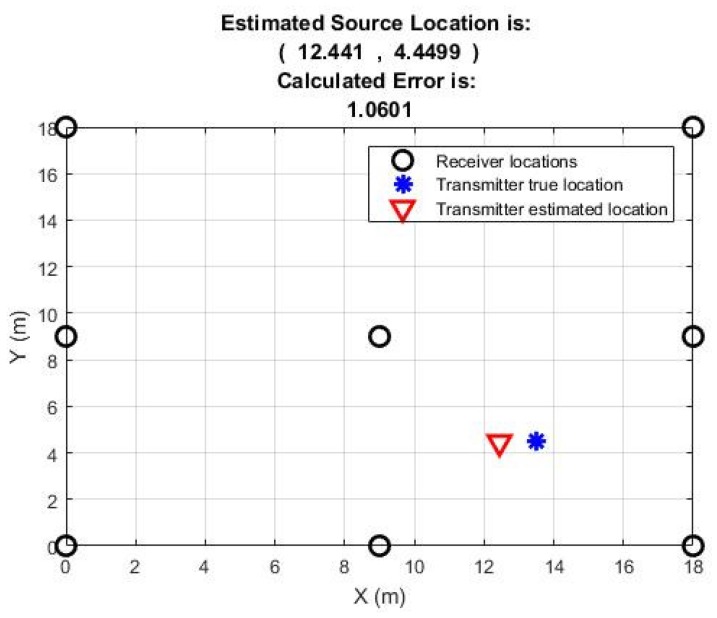
Position 1 result of the estimated source location with eight receiving nodes.

Table 3 shows that results have improved significantly in terms of localization accuracy and PLE optimizations. For all nine positions, the PLE values are from 1.55 to 3.35, with the majority between 2 to 3. The localization error for six position is less than two meters. For three positions it is between 2 to 3 m. This is an indication of how scalability can enhance the localization accuracy. The localization accuracy also means that PLE values are much closer to the average value of the free space propagation environment, i.e., 2 in this case. For position 1 the results are shown in Figure 10.

For both field scenarios, the mean estimated error for the proposed algorithm can be summarized in Table 4 below:

Table 4 shows that mean error was reduced from 1.80 m to 1.76 m, i.e., 0.04 m better localization accuracy with the addition of a single node. This implies that RSS based localization is a technique with better properties in PD localization due to its capability to offer scalability at any given time without any modifications in the overall system configuration except the addition of a receiving node.

### 3.2. Performance Evaluation of the Proposed Algorithm in Terms of Localization Error and PLE Optimization When Seven Measurement Sensors were Used

The performance of the proposed algorithm was evaluated with ratio and search and least squares (LS) algorithms. All three algorithms use RSS for localization under an anonymous environment i.e., having no prior information of source transmitted power and path loss exponent. The performance of the algorithms was evaluated in terms of localization error and optimized values of the path loss exponent in two field-trial scenarios. The least squares algorithm is quite simple and does not optimize the path loss exponent. The proposed algorithm and ratio and search algorithms both optimize the value of path loss exponent within a given range. When seven measurement nodes were used, the comparison of true versus estimated locations between the three algorithms is shown in Table 5.

Estimated location values shown in Table 5 above are rounded to two decimal places. Comparing the true versus estimated locations, it is evident that all three algorithms estimate the coordinates of the source within reasonable accuracy. For the majority of the locations, the estimated versus the true locations seem reasonably close. A comparison of error calculation and PLE for each position when seven measurement sensors are used is shown in Table 6. 

**Figure 11:**
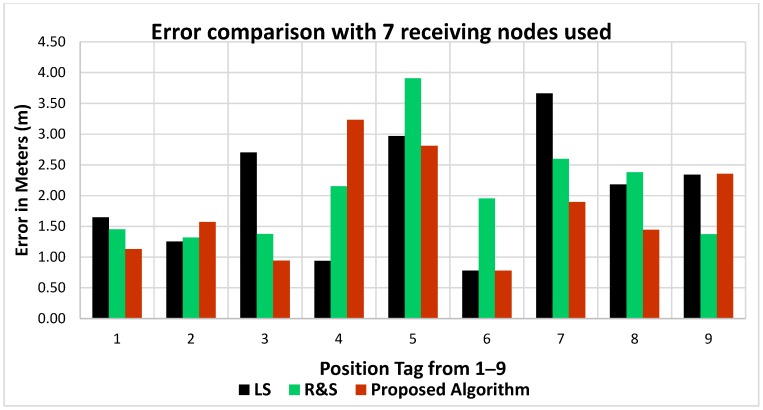
Error comparison with seven measurement sensors used.

Table 6 shows the error comparison for each position as well as the optimized value of the PLE. The results show that for the majority of the positions, the proposed algorithm offers better accuracy and lower mean error. An error comparison between the algorithms can be seen in Figure 11, where seven measurement sensors were used.

From the above comparison, it is evident that the proposed algorithm offers the least error when compared with the other algorithms and that the path loss exponent estimation accuracy has also improved for nearly all positions.

### 3.3. Performance Evaluation of the Proposed Algorithm in Terms of Localization Error and PLE Optimization When Eight Measurement Sensors were Used

To evaluate the performance of the proposed algorithm further, a node is added in the receiving system, and this time eight measurement sensors are used. A comparison between the estimated and true locations has been made and error is computed for each position as well as the optimized PLE.

When eight measurement sensors are used, the comparison between the true and estimated locations for each algorithm is shown in Table 7.

Table 7 results are based on eight receiving nodes. With the addition of a single node to the receiving system, the source location estimations have improved for the majority of positions for the ratio and search and the proposed algorithms. A comparison of error calculation and PLE for each position when eight measurement sensors are used is shown in Table 8.

**Figure 12:**
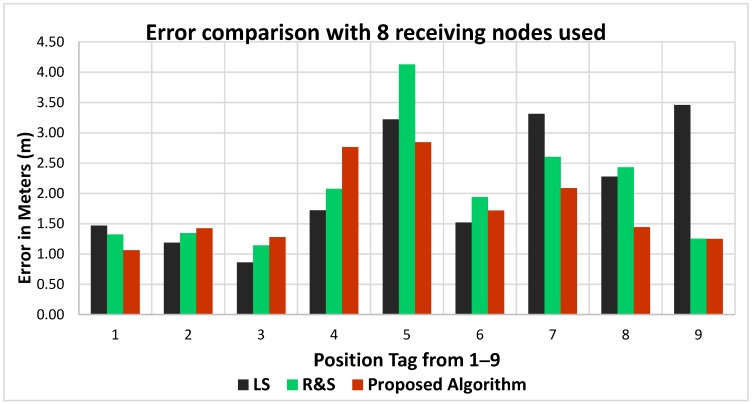
Error comparison with eight measurement sensors used.

The least squares algorithm performance has improved for positions 1, 2 and 3, however, the mean error has slightly increased although not to a great extent. This is mainly due to the fact that the least squares algorithm does not optimize the PLE. For the ratio and search and the proposed algorithm, the results have improved in terms of localization accuracy for the majority of the positions with less mean error for all nine positions. For ratio and search algorithm the mean error has slightly improved i.e., from 2.06 to 2.03 an improvement of 0.03 m. For the proposed algorithm, the mean error has improved from 1.80 m to 1.76 m i.e., an improvement of 0.04 m. This shows that by increasing the number of nodes, the overall location accuracy of the PD source estimation is improved. An error comparison between the algorithms can be seen in Figure 12, where eight measurement sensors were used.

Results shown in Table 8 are much improved when compared with Table 7 for ratio and search and proposed algorithms. For individual locations, the accuracy has improved when compared with seven sensors used. For the proposed algorithm, the PLE values seem to be more realistic for most of the nine positions.

## 4. Conclusions

PD location estimation was performed by using RSS and a novel algorithm has been proposed. Practical measurements were conducted and signals received were the voltage level recorded. Voltage levels were converted into power (d*Bm*) as input to the location algorithm. The location estimation was performed by converting the received signal into distance and (x,y) coordinates of the source were estimated for nine different positions in two field-trial scenarios. The algorithm did not have any prior information about the source transmitted power and the path loss exponent. An initial path loss exponent value of 2 was chosen and then it was optimized using measured spread. The proposed algorithm was compared to least squares and ratio and search algorithms. The results show that RSS based localization is a plausible technique and the proposed algorithm offers better results in terms of localization accuracy and path-loss exponent estimation as the number of receiving nodes are increased from seven to eight for the majority of positions. The proposed algorithm optimizes the path loss exponent, however, this optimized value is the same for all node pairs. In a real environment, path loss exponent may vary. This is one of the limitations of the proposed algorithm as well as the reference algorithms.

## Figures and Tables

**Table 1:** Received signal power in d*Bm*.

Source Position	Node 1	Node 2	Node 3	Node 4	Node 5	Node 6	Node 7	Node 8
Position 1	−12.49	−12.69	−12.32	−4.35	−4.56	−8.98	−3.58	−6.27
Position 2	−13.10	−14.38	−5.72	−4.05	−10.36	−6.87	−4.88	−14.33
Position 3	−1.91	−12.19	−4.43	−7.10	−11.99	−13.42	−12.44	−17.48
Position 4	−9.23	−2.56	−11.70	−5.16	−5.29	−13.33	−14.11	−18.50
Position 5	−11.91	−11.17	−10.47	2.30	−3.49	−9.69	−8.92	−10.50
Position 6	−9.41	−13.18	−3.07	1.28	−9.82	−9.75	−9.01	−14.69
Position 7	−6.10	−8.90	−8.53	−0.40	−7.76	−12.70	−12.78	−17.73
Position 8	−11.19	−6.56	−12.18	1.29	0.68	−12.13	−10.01	−13.58
Position 9	−18.49	−19.26	−14.94	−13.44	−15.21	−2.62	−4.96	−12.84

**Table 2:** PD source location estimation with seven sensors used, proposed method.

Source Position	True Location		Estimated Location		Error (m)	Optimum PLE α
	X (m)	Y (m)	X (m)	Y (m)		
Position 1	13.50	4.50	12.37	4.49	1.13	1.75
Position 2	4.50	4.50	5.99	5.02	1.57	2.15
Position 3	4.50	13.50	5.41	13.76	0.94	3.45
Position 4	13.50	13.50	10.94	15.48	3.23	4.25
Position 5	10.00	6.00	12.65	6.95	2.81	1.60
Position 6	6.00	8.00	6.60	8.50	0.78	2.05
Position 7	8.00	12.00	7.89	13.89	1.90	3.30
Position 8	12.00	10.00	13.16	10.86	1.45	2.60
Position 9	4.5.0	−4.50	3.14	−6.43	2.36	2.75

**Table 3:** PD source location estimation with eight sensors used, proposed method.

Source Position	True Location		Estimated Location		Error (m)	Optimum PLE α
	X (m)	Y (m)	X (m)	Y (m)		
Position 1	13.50	4.50	12.44	4.45	1.06	1.75
Position 2	4.50	4.50	5.73	5.22	1.43	2.20
Position 3	4.50	13.50	4.12	14.72	1.28	2.80
Position 4	13.50	13.50	11.35	15.24	2.77	3.55
Position 5	10.00	6.00	12.58	7.20	2.85	1.55
Position 6	6.00	8.00	5.63	9.68	1.72	2.50
Position 7	8.00	12.00	8.95	13.86	2.08	2.95
Position 8	12.00	10.00	13.18	10.83	1.44	2.70
Position 9	4.5.0	−4.50	3.97	−5.63	1.24	2.85

**Table 4:** Mean error comparison for different arrangements of sensors.

	With 7 Sensors	With 8 Sensors
Mean Error (m)	1.80	1.76

**Table 5:** Comparison of estimated versus true location with seven measurement sensors.

Source Position	Actual Locations		LS Estimated Locations		R&S Estimated Locations		Proposed Algorithm Estimated Locations	
	X (m)	Y (m)	X (m)	Y (m)	X (m)	Y (m)	X (m)	Y (m)
Position 1	13.50	4.50	11.88	4.77	12.08	4.82	12.37	4.49
Position 2	4.50	4.50	5.55	5.19	5.26	5.58	5.99	5.02
Position 3	4.50	13.50	7.07	12.66	4.33	14.87	5.41	13.76
Position 4	13.50	13.50	12.60	13.77	11.63	14.56	10.94	15.48
Position 5	10.00	6.00	11.99	8.20	12.64	8.88	12.65	6.95
Position 6	6.00	8.00	5.80	8.75	5.40	9.86	6.60	8.50
Position 7	8.00	12.00	11.64	12.45	7.86	14.59	7.89	13.89
Position 8	12.00	10.00	13.35	11.71	12.57	12.31	13.16	10.86
Position 9	4.50	−4.50	3.78	−6.73	3.46	−5.40	3.14	−6.43

**Table 6:** Error comparison for seven measurement sensors.

Source Position	LS Error (m)	LS PLE	R&S Error (m)	R&S PLE	Proposed Algorithm Error (m)	Proposed Algorithm PLE
Position 1	1.65	2	1.45	1.65	1.13	1.75
Position 2	1.26	2	1.32	2.15	1.57	2.15
Position 3	2.70	2	1.38	3.2	0.94	3.45
Position 4	0.94	2	2.15	1.50	3.23	4.25
Position 5	2.97	2	3.91	1.50	2.81	1.60
Position 6	0.78	2	1.95	2.50	0.78	2.05
Position 7	3.66	2	2.60	3.20	1.90	3.30
Position 8	2.18	2	2.38	2.80	1.45	2.60
Position 9	2.34	2	1.37	2.80	2.36	2.75
Mean Error	2.05		2.06		1.80	

**Table 7:** Comparison of estimated versus true locations with eight measurement sensors.

Source Position	Actual Locations		LS Estimated Locations		R&S Estimated Locations		Proposed Algorithm Estimated Locations	
	X (m)	Y (m)	X (m)	Y (m)	X (m)	Y (m)	X (m)	Y (m)
Position 1	13.50	4.50	12.04	4.69	12.21	4.77	12.44	4.45
Position 2	4.50	4.50	5.41	5.27	5.38	5.52	5.73	5.22
Position 3	4.50	13.50	4.91	14.26	4.33	14.63	4.12	14.72
Position 4	13.50	13.50	11.89	14.11	11.68	14.50	11.35	15.24
Position 5	10.00	6.00	11.81	8.67	12.55	9.25	12.58	7.20
Position 6	6.00	8.00	5.15	9.26	5.45	9.86	5.63	9.68
Position 7	8.00	12.00	8.80	15.21	7.89	14.60	8.95	13.86
Position 8	12.00	10.00	13.32	11.86	12.53	12.37	13.18	10.83
Position 9	4.50	−4.50	3.91	−7.91	3.91	−5.60	3.97	−5.63

**Table 8:** Error and PLE comparison for eight measurement sensors.

Source Position	LS Error (m)	LS PLE	R&S Error (m)	R&S PLE	Proposed Algorithm Error (m)	Proposed Algorithm PLE
Position 1	1.47	2	1.32	1.65	1.06	1.75
Position 2	1.19	2	1.35	2.15	1.43	2.20
Position 3	0.86	2	1.15	3.2	1.28	2.80
Position 4	1.72	2	2.08	1.50	2.77	3.55
Position 5	3.22	2	4.13	1.50	2.85	1.55
Position 6	1.52	2	1.94	2.50	1.72	2.50
Position 7	3.31	2	2.60	3.20	2.08	2.95
Position 8	2.28	2	2.43	2.80	1.44	2.70
Position 9	3.46	2	1.25	2.80	1.24	2.85
Mean Error	2.11		2.03		1.76	
